# Association Between Adherence to Evidence-Based Practices for Treatment of Patients With Traumatic Rib Fractures and Mortality Rates Among US Trauma Centers

**DOI:** 10.1001/jamanetworkopen.2020.1316

**Published:** 2020-03-02

**Authors:** Christopher J. Tignanelli, Alexander Rix, Lena M. Napolitano, Mark R. Hemmila, Sisi Ma, Erich Kummerfeld

**Affiliations:** Department of Surgery, University of Minnesota Medical School, Minneapolis; Department of Surgery, North Memorial Health Hospital, Robbinsdale, Minnesota; Institute for Health Informatics, University of Minnesota Academic Health Center, Minneapolis; Institute for Health Informatics, University of Minnesota Academic Health Center, Minneapolis; Department of Surgery, University of Michigan, Ann Arbor; Department of Surgery, University of Michigan, Ann Arbor; Institute for Health Informatics, University of Minnesota Academic Health Center, Minneapolis; Institute for Health Informatics, University of Minnesota Academic Health Center, Minneapolis

## Abstract

**IMPORTANCE:**

Rib fractures are sustained by nearly 15% of patients who experience trauma and are associated with significant morbidity and mortality. Evidence-based practice (EBP) rib fracture management guidelines and treatment algorithms have been published. However, few studies have evaluated trauma center adherence to EBP or the clinical outcomes of each practice within a national cohort.

**OBJECTIVE:**

To examine adherence to 6 EBPs for rib fractures across US trauma centers and the association with in-hospital mortality.

**DESIGN, SETTING, AND PARTICIPANTS:**

A retrospective cohort study was conducted from January 1, 2007, to December 31, 2014, of 777 US trauma centers participating in the National Trauma Data Bank. A total of 625 617 patients (age, ≥16 years) were evaluated. Patients without rib fractures and those with no signs of life or institutions with poor data quality were excluded. Data analysis was performed from January 1, 2007, to December 31, 2014.

**MAIN OUTCOMES AND MEASURES:**

Six EBPs were defined: (1) neuraxial blockade, (2) intensive care unit admission, (3) pneumatic stabilization, (4) chest computed tomographic scans for older adults (≥65 years) with 3 or more rib fractures, (5) surgical rib fixation for flail chest, and (6) tube thoracostomy placement for hemothorax and/or pneumothorax. Multiple imputation was used to account for missing data. Patients were propensity score matched in a 1:1 fashion based on demographic characteristics; injury severity parameters, including the Injury Severity Score (range, 0–75; higher scores indicate more severe injuries); and comorbidities. Logistic regression was used to determine the association of each practice with all-cause in-hospital mortality.

**RESULTS:**

Of the 625 617 patients with rib fractures included in this analysis, 456 196 patients (73%) were white and 432 229 patients (69%) were male; the median age of the patients was 51 (interquartile range, 37–65) years, and the mean (SD) Injury Severity Score was 18.3 (11.1). The mean (SD) number of rib fractures was 4.2 (2.6). On univariate analysis, patients treated at verified level I trauma centers were more likely to receive 5 or 6 EBPs (all but pneumatic stabilization). Of those who met eligibility, only 4578 of 111 589 patients (4%) received neuraxial blockade, 46 456 of 111 589 patients (42%) were admitted to the intensive care unit, 3302 of 24 319 patients (14%) received surgical rib fixation, 1240 of 111 589 patients (1%) received pneumatic stabilization, 109 160 of 258 334 patients (42%) received tube thoracostomy, and 32 405 of 81 417 patients (40%) received chest computed tomographic scans. Three EBPs were associated with decreased mortality: neuraxial blockade (odds ratio [OR], 0.64; 95% CI, 0.51–0.79; *P* < .001) for patients aged 65 years or older with 3 or more rib fractures, surgical rib fixation (OR, 0.13; 95% CI, 0.01–0.18; *P* < .001), and intensive care unit admission (OR, 0.93; 95% CI, 0.86–1.00; *P* = .04) for patients aged 65 years or older with 3 or more rib fractures. Pneumatic stabilization (OR, 1.71; 95% CI, 1.25–2.35; *P* < .001) and chest tube placement (OR, 1.27; 95% CI, 1.21–1.33; *P* < .001) were associated with increased mortality in older patients with 3 or more rib fractures. On multivariable analysis, insurance status, race/ethnicity, injury severity, hospital bed size, and trauma center verification level were associated with receiving EBPs for rib fractures.

**CONCLUSIONS AND RELEVANCE:**

Significant variation appears to exist in the delivery of EBPs for rib fractures across US trauma centers. Three EBPs were associated with reduced mortality, but EBP adherence was poor. Multiple factors, including trauma center verification level, appear to be associated with patients receiving EBPs for rib fractures.

## Introduction

Chest trauma is involved in nearly one-third of trauma admissions worldwide, accounting for 25% of trauma-related deaths, with rib fractures sustained by nearly 15% of patients who experience trauma and in approximately two-thirds of chest trauma.^1,2^ Rib fractures are associated with significant morbidity (48% complication rate) and mortality (22% for older adults).^3,4^ Of patients aged 60 years or older who die from chest trauma, 55% have no injury worse than rib fractures.^5^

The initiation of the Michigan Trauma Quality Improvement Program and the American College of Surgeons (ACS) Trauma Quality Improvement Program occurred in response to concerns of variation in clinical outcomes for injured patients.^6–8^ In addition, national societies have developed practice management guidelines, standard operating procedures, and treatment algorithms since the 1990s in an attempt to better inform the delivery of trauma care, improve adherence to evidence-based practices (EBPs), and reduce variation in clinical outcomes.^9^ Studies suggest that, if all hospitals delivered the highest quality of care to patients who have experienced trauma, an estimated 167 746 lives could potentially be saved over 9 years.^10^

Despite these efforts, few studies have evaluated trauma center adherence to rib fracture EBP nationally or factors associated with adherence. A better understanding of patient and trauma center factors associated with EBP adherence is necessary. Such knowledge may facilitate development of interventions aimed to improve EBP. Thus, the purpose of this study was to evaluate US trauma center adherence to and the outcomes associated with 6 EBPs for patients with rib fractures treated at US trauma centers between January 1, 2007, and December 31, 2014.

## Methods

The data source for this cohort study was the ACS National Trauma Data Bank (NTDB),^11^ which includes patient- and hospital-level data on traumatic injuries and clinical outcomes for more than 900 trauma centers. We limited our analysis to years after 2006 because of data quality improvements implemented in 2007.

This study was approved by the University of Minnesota Institutional Review Board, which provided a waiver of consent for this study because the data in the NTDB are deidentified. This study followed the Strengthening the Reporting of Observational Studies in Epidemiology (STROBE) reporting guideline for cohort studies.^12^

We obtained patient-level data from the NTDB from January 1, 2007, through December 31, 2014. The inclusion criteria were age 16 years or older and presence of 1 or more rib fractures (*International Classification of Diseases, Ninth Revision* [*ICD-9*] codes 807.00–807.18) or flail chest (*ICD-9* code 807.4).

We aimed to exclude centers that did not consistently code common procedures. We identified and excluded centers where no patient underwent the 2 most commonly coded operative procedures in the NTDB, namely, open reduction and internal fixation of the femur and internal fixation of the tibia (*ICD-9* procedure codes 79.35–79.36).^13^ In addition, we excluded patients with no signs of life at initial evaluation in the emergency department: systolic blood pressure, 0 mm Hg; pulse, 0 beats/min; Glasgow Coma Scale score, 3; or unknown status (ie, we were unable to determine whether they were dead on arrival or died during their hospitalization). We also excluded patients who were not admitted from the emergency department or were missing the emergency department disposition variable.

We defined 6 EBPs for patients with rib fractures based on national trauma guidelines.^9,14^ We chose practices with varying levels of evidence. The recommendations evaluated were the following:

- EBP1: Neuraxial (epidural or paravertebral) block placement for older adults (aged ≥65 years) with 3 or more rib fractures who were not transferred to another facility for definitive management.^15–18^ Epidural analgesia was defined by *ICD-9* codes 3.90 and 3.91, and paravertebral block was defined by *ICD-9* codes 4.80 and 4.81.
- EBP2: Intensive care unit (ICU) admission for older adults (aged ≥65 years) with 3 or more rib fractures who were not transferred to another facility for definitive management.^9,19,20^
- EBP3: Surgical rib fixation for patients with flail chest who were not transferred to another facility for definitive management.^14,21–24^
- EBP4: Pneumatic stabilization via noninvasive ventilation for older adults (aged ≥65 years) with 3 or more rib fractures who were not transferred to another facility for definitive management.^16,25–28^ Noninvasive ventilation was defined by *ICD-9* codes 93.90 and 93.91.
- EBP5: Tube thoracostomy placement for patients with a hemothorax, pneumothorax, or a combination of the conditions (ie, hemothorax/pneumothorax).^29–31^ Hemothorax/pneumothorax was defined by the *ICD-9* codes 860.0 to 860.5. Chest tube placement was identified via *ICD-9* procedure codes 34.01, 34.02, 34.04, and 34.09. Current practice recommends treatment for all patients with a hemothorax, nonoccult pneumothorax, or the combination. We were unable to differentiate between occult vs nonoccult pneumothorax owing to limitations in *ICD-9* coding.
- EBP6: Chest computed tomographic (CT) scans for older patients (aged ≥65 years) with 3 or more rib fractures who were not transferred into or out of the facility.^32–34^ Chest radiographs alone can miss up to 75% of rib fractures seen on CT scans—approximately 35% of patients will have a change in clinical management based on chest CT scan findings.^32^ Computed tomographic scanning of the chest was identified via *ICD-9* procedure codes 87.41 and 87.42.

### Statistical Analysis

Statistical analysis was conducted from January 1, 2007, to December 31, 2014. To report measures of central tendency in univariate analysis, means (SDs) for continuous variables with a normal distribution or medians (interquartile ranges [IQRs]) for continuous variables with a skewed distribution were reported. To compare the distribution of mortality across demographic variables, χ^2^ for categorical variables, *t* test for normally distributed continuous variables, and Wilcoxon rank sum test for not normally distributed continuous variables were used.

To evaluate the association of EBP adherence with in-hospital mortality, we considered all patients who met a given EBP criterion and compared the mortality outcome between patients who received the EBP with those who did not receive the care recommended by the EBP. To adjust for confounding bias, we constructed a matched case and control cohort in a 1:1 fashion by propensity score matching.^35^ A double propensity score adjustment was undertaken using the same covariates in both the treatment and outcome model.^36^ This method was used for 2 reasons: to account for residual bias from imperfect matching and because the adjustment covariates are related to the outcome (mortality) and thus should be included to reduce the SE of the estimated association.^37^ The propensity score of receiving EBPs was computed using a logistic regression model with the following covariates: age, sex, race/ethnicity, insurance status, drug use information, Injury Severity Score (ISS), which is a validated tool to assess trauma severity (range, 0–75; higher number represents more severe injury),^38^ motor component of the Glasgow Coma Scale,^39^ emergency department–reported most aberrant vital signs (systolic blood pressure, respiratory rate, and heart rate), number of rib fractures, presence of flail chest, hemothorax/pneumothorax, and comorbidities (ie, alcoholism, congestive heart failure, cirrhosis, esophageal varices, and disseminated cancer). These covariates were chosen owing to their status as risk factors that were associated with mortality probability. Continuous variables were not categorized. The primary outcome of interest was all-cause in-hospital mortality. The Glasgow Coma Scale motor score was used owing to being comparable with the total Glasgow Coma Scale score.^40^ Patients with an ISS less than or equal to 10 were excluded to better assess the potential treatment outcome in high-risk populations. The resulting model was used to generate a propensity score from 0 to 1 for each patient, representing their propensity of receiving the EBP. The cases and controls were matched on their propensity score with nearest neighbor matching with a caliper of 0.01. A nearest neighbor propensity score matching with replacement was done for EBP2 because the matched propensity scores were substantially different between the case and control populations when matching without replacement.^41^ All matched cohorts were evaluated for balance between the case and control groups regarding each of the potential confounding factors. The association of EBPs with mortality was quantified in the matched cohorts using logistic regression.

To address missing data, multiple imputation was performed in R, version 3.5.0 (R Project for Statistical Computing) using the package multivariate imputation by chained equations, version 3.3.0,^19^ to impute the missing values. Five imputed data sets were created. Convergence was assessed by comparing the mean (SD) of imputed variables as a function of the number of iterations across imputed data sets.

The MatchIt, version 3.0.2, package was used to perform propensity score matching.^42^ All mentioned covariates were included with the exception of the number of rib fractures or flail chest. Propensity score matches were assessed by examination of the mean balance improvement and univariate statistical tests (*t* test and χ^2^ analysis) and visually via quantile-to-quantile plots for each EBP on each imputed data set.

For each EBP, logistic regressions with the same model as the propensity scoring were performed on the 5 matched imputed data sets. These regressions were pooled to generate an estimate and SE of each EBP’s outcome. In addition, a logistic model containing only the treatment and intercept was fit and pooled in a similar manner. Given that replacement was used to generate a propensity score–matched data set for EBP2, the R survey package was used to perform weighted distributional tests and weighted logistic regression for that EBP. Statistical analysis was conducted using 2-tailed testing, with findings considered significant at α = .05.

## Results

**Figure 1.**
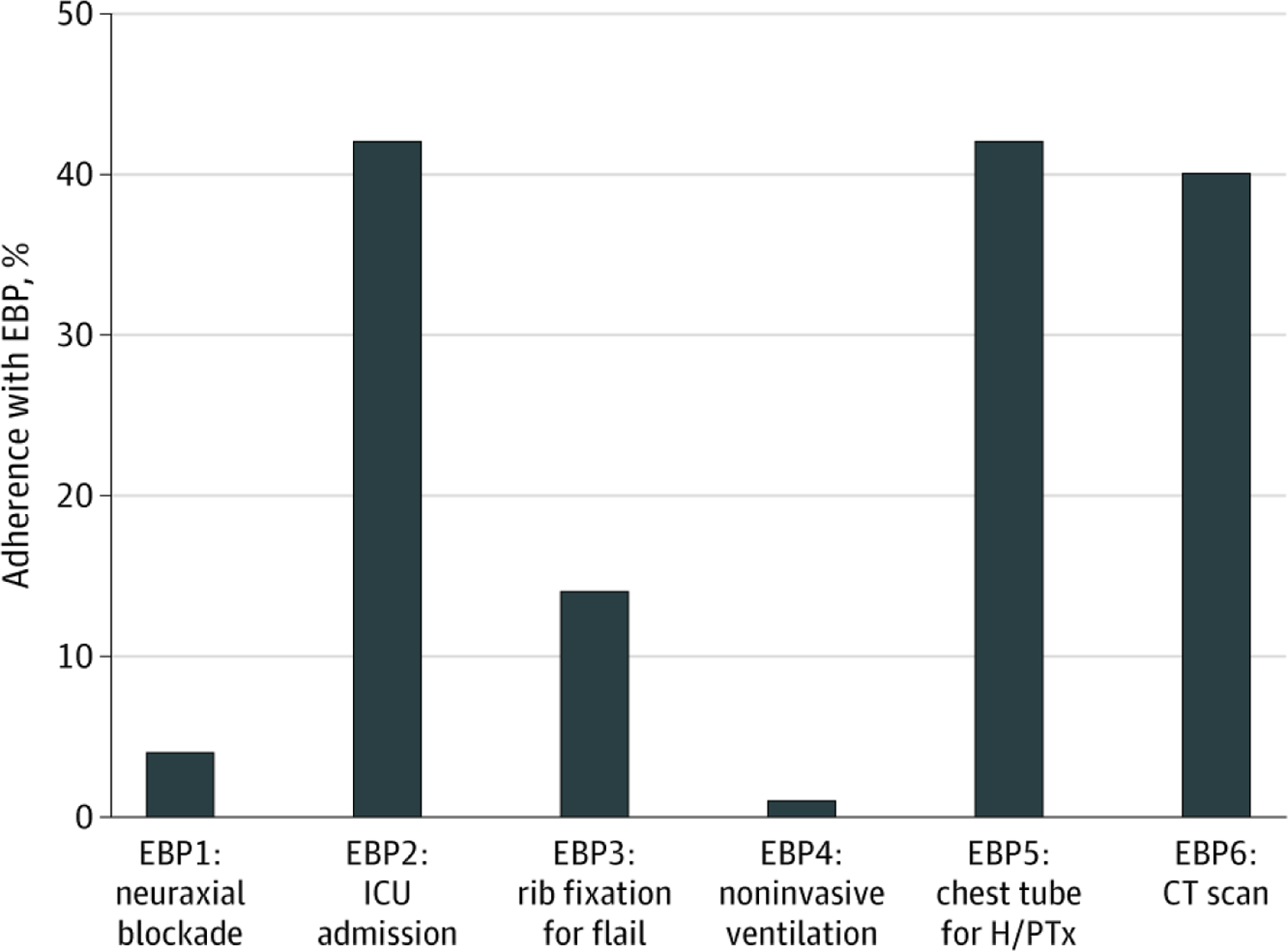
Overall Adherence to 6 Evidence-Based Practices (EBPs) for Patients With Rib Fractures ICU indicates intensive care unit; H/PTx, hemothorax/pneumothorax; and CT, computed tomographic.

**Figure 2.**
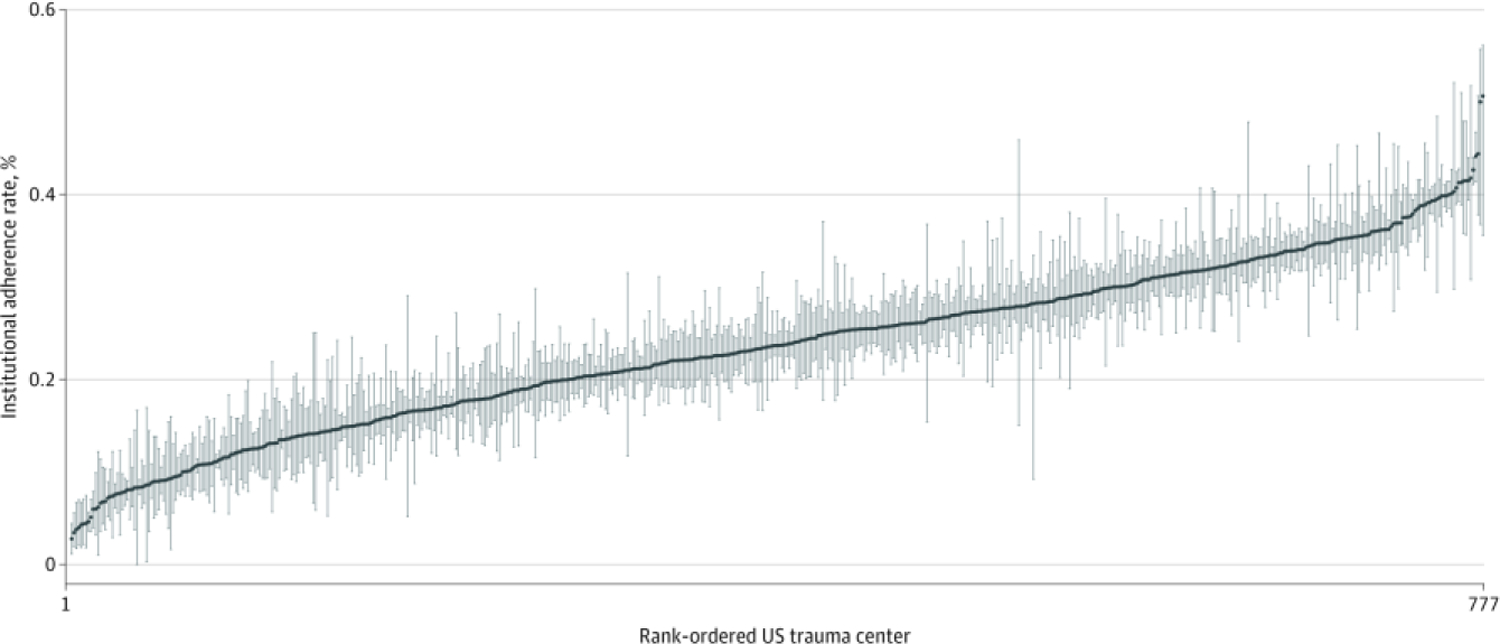
Variation of Rib Fracture Evidence-Based Practice Across US Trauma Centers Graph represents institutional adherence with evidence based practice. The blue line represents the mean adherence, and the blue bars represent bootstrapped (replications = 200) 95% confidence interval per trauma center.

A total of 625 617 patients from 777 US trauma centers met the inclusion criteria (eFigure 1 in the Supplement). The median age of the patients was 51 (IQR, 37–65) years, 456 196 patients (73%) were white, 432 229 patients (69%) were male, and the mean (SD) ISS was 18.3 (11.1). The patients had a mean of 4.2 (2.6) rib fractures. Adherence to EBP was poor overall: only 4578 of 111 589 patients (4%) received neuraxial blockade, 46 456 of 111 589 patients (42%) were admitted to the intensive care unit, 3302 of 24 319 patients (14%) received surgical rib fixation, 1240 of 111 589 patients (1%) received pneumatic stabilization, 109 160 of 258 334 patients (42%) received tube thoracostomy, and 32 405 of 81 417 patients (40%) received chest CT scans (Figure 1). At the hospital level, significant variation in EBP adherence was noted across US trauma centers (Figure 2), with an institutional adherence rate ranging from 2.79% to 50.6%. On univariate analysis, patients treated at verified level I trauma centers were more likely to receive 5 of the 6 evaluated EBPs (all but pneumatic stabilization). For example, older patients with 3 or more rib fractures were more likely to be admitted to the ICU at ACS-verified level I trauma centers (47%) compared with ACS-verified level II trauma centers (39%) and ACS-verified level III trauma centers (17%) (*P* < .001).

EBP1 adherence vs nonadherence was more common in patients with the following characteristics: younger (median age, 75 [IQR, 69–82] vs 76 [IQR, 70–82] years, *P* = .006), white (4009 [90.6%] vs 89 429 [87.2%], *P* < .001), higher ISS (median score, 16.0 [IQR, 10.0–22.0] vs 14.0 [IQR, 9.0–21.0], *P* < .001), increased median number of rib fractures (7.0 [IQR, 5.0–9.0] vs 5.0 [IQR, 4.0–7.0], *P* < .001), associated hemothorax/pneumothorax (2261 [49.4%] vs 36 057 [33.7%], *P* < .001), treatment at an ACS-verified level I trauma center (1831 [40.0%] vs 37 814 [35.3%], *P* < .001), and injured more recently (993 [21.7%] vs 20 661 [19.3%], *P* < .001) (Table 1).

EBP2 adherence vs nonadherence was more common in patients with the following characteristics: younger (median age, 75 [IQR, 70–82] vs 76 [IQR, 69–83] years, *P* < .001), male (27 626 [59.6%] vs 35 061 [54.0%], *P* < .001), private insurance (14 804 [34.9%] vs 17 952 [30.1%], *P* < .001), higher ISS (median score, 17.0 [IQR, 13.0–25.0] vs 12.0 [IQR, 9.0–17.0], *P* < .001), lower mean systolic blood pressure (139.0 [IQR,118.0–159.0] vs 144.0 [IQR, 126.0–162.0] mm Hg, *P* < .001), more rib fractures (median, 6.0 [4.0–9.0] vs 5.0 [IQR, 3.0–6.0], *P* < .001), an associated hemothorax/pneumothorax (19 458 [41.9%] vs 18 860 [29.0%], *P* < .001), and treatment at an ACS-verified level I trauma center (18 615 [40.1%] vs 21 030 [32.3%], *P* < .001) (Table 1).

EBP3 adherence was more common in patients with the following characteristics: younger (median age, 52 [IQR, 43–62] vs 54 [IQR, 44–66] years, *P* < .001), private insurance (1850 [60.6%] vs 10 193 [53.7%], *P* < .001), an associated hemothorax/pneumothorax (2763 [83.7%] vs 15 747 [74.9%], *P* < .001), treatment at an ACS-verified level I trauma center (1512 [45.8%] vs 8409 [40.0%], *P* < .001), and injured more recently (696 [21.1%] vs 3285 [15.6%], *P* < .001) (Table 2).

EBP4 adherence vs nonadherence was more common in patients with the following characteristics: male (777 [62.7%] vs 61 910 [56.3%], *P* < .001), white (1090 [91.3%] vs 92 348 [87.3%], *P* < .001), Medicare insurance (768 [66.3%] vs 63 443 [62.9%], *P* < .001), higher ISS (median score, 16.0 [IQR, 10.0–22.0] vs 14.0 [IQR, 9.0–21.0], *P* < .001), tachypnea (median, 20.0 [IQR, 18.0–22.0] vs 18.0 [IQR, 16.0–20.0] breaths/min, *P* < .001), an associated hemothorax/pneumothorax (554 [44.7%] vs 37 764 [34.2%], *P* < .001), treatment at a nondesignated trauma center (620 [50.0%] vs 47 111 [42.7%], *P* < .001), and injured more recently (291 [23.5%] vs 21 363 [19.4%], *P* < .001) (Table 2).

EBP5 adherence vs nonadherence was more common in patients with the following characteristics: younger (median age, 48 [IQR,31–61] vs 48 [IQR, 32–61] years, *P* < .001), male (81 590 [74.8%] vs 105 136 [70.5%], *P* < .001), black (vs 13 909 [13.2%] vs 14 150 [9.8%], *P* < .001), higher ISS (median score, 21.0 [IQR, 14.0–29.0] vs 17.0 [IQR, 12.0–24.0], *P* < .001), lower systolic median blood pressure (129.0 [IQR, 109.0–146.0] vs 134.0 [IQR, 118.0–150.0] mm Hg, *P* < .001), tachypnea (median, 20.0 [IQR, 16.0–24.0] vs 18.0 [IQR, 16.0–22.0] breaths/min, *P* < .001), and more rib fractures (median, 5.0 [IQR, 2.0–8.0] vs 4.0 [IQR, 2.0–7.0], *P* < .001) (Table 3).

EBP6 adherence vs nonadherence was more common in patients with the following characteristics: younger (median age, 76 [IQR, 69–82] vs 76 [IQR, 70–83] years, *P* < .001), male (18 124 [56.0%] vs 26 629 [54.5%], *P* < .001), white (27 481 [87.6%] vs 39 565 [84.8%], *P* < .001), private insurance (10 681 [34.9%] vs 13 808 [31.2%], *P* < .001), higher ISS (median score, 14.0 [IQR, 9.0–20.0] vs 13.0 [IQR, 9.0–21.0], *P* < .001), an associated hemothorax/pneumothorax (11 422 [35.2%] vs 14 853 [30.3%], *P* < .001), treatment at an ACS-verified level I trauma center (10 487 [33.5%] vs 14 688 [30.0%], *P* < .001), and injured more recently (8273 [25.5%] vs 7320 [14.9%], *P* < .001) (Table 3).

The association with each EBP on in-hospital mortality was then evaluated using double propensity score adjusment.^36^ eTable 1 in the Supplement displays the sample sizes in the prematching and postmatching cohorts for each EBP. Propensity scores were optimally balanced between cohorts (eFigures 2–7 in the Supplement). Adherence to 3 EBPs was associated with reduced mortality: epidural placement in patients aged 65 years or older with 3 or more rib fractures (odds ratio [OR], 0.64; 95% CI, 0.51–0.79; *P* < .001), rib fixation for flail chest (OR, 0.13; 95% CI, 0.01–0.18; *P* < .001), and ICU admission for patients aged 65 years or older with 3 or more rib fractures (OR, 0.93; 95% CI, 0.86–1.00; *P* = .04). Noninvasive ventilation was associated with increased mortality in patients aged 65 years or older with 3 or more rib fractures (OR, 1.71; 95% CI, 1.25–2.35; *P* < .001). Chest tube placement was also associated with increased mortality for patients with hemothorax/pneumothorax (OR, 1.27; 95% CI, 1.21–1.33; *P* < .001). Findings of CT scans performed when indicated were not associated with mortality (OR, 0.98; 95% CI, 0.91–1.05; *P* = .55).

In addition, we evaluated patient- and hospital-level factors associated with receiving an EBP using the logistic model from which propensity scores were derived (eTable 2 in the Supplement). In general, for 2 of the 3 EBPs associated with improved survival, nonwhite individuals were less likely to receive EBP (neuraxial blockade: Hispanic ethnicity [OR, 0.49; 95% CI, 0.39–0.62; *P* < .001] and Asian race [OR, 0.58; 95% CI, 0.43–0.79; *P* < .001] compared with white race and rib fixation: black race [OR, 0.84; 95% CI, 0.73–0.98; *P* = .02] and Hispanic ethnicity [OR, 0.80; 95% CI, 0.69–0.93; *P* = .003]). For all 3 EBPs associated with improved survival, underinsured patients were less likely to receive EBP. For all EBPs, the number of rib fractures or presence of hemothorax and/or pneumothorax was associated with increased odds of receiving EBP (eTable 2 in the Supplement). In addition, for the 3 EBPs associated with improved survival, patients treated at non–ACS-verified level I trauma centers were less likely to receive EBPs. Hospital bed size was also significantly associated with receiving EBP. For example, each additional hospital bed was associated with an increased odds of recieving neuraxial blockade in patients aged 65 years or older with 3 or more rib fractures (OR, 1.0003; 95% CI, 1.0001–1.0005; *P* < 0.001).

## Discussion

In this study, we identified significant variation in adherence to 6 EBPs for patients with rib fractures. We identified a low rate of adherence to neuraxial blockade, rib fixation, and ICU admission EBPs despite evidence-based national guideline recommendations. Forty percent of the eligible patients received a chest CT scan, and 42% of the patients with hemothorax/pneumothorax received tube thoracostomy. The findings also showed low use of pneumatic stabilization for elderly patients with 3 or more rib fractures. Significant variation in EBPs was noted across trauma centers, with institutional adherence ranging from 2.8% to 50.6%. We identified that only 3 EBPs were associated with an inpatient survival benefit: neuraxial blockade, rib fixation, and ICU admission. Two EBPs were associated with increased mortality: tube thoracostomy and pneumatic stabilization. The EBPs associated with survival benefit were all significantly less likely to be performed at undesignated trauma centers. Practices with high resource burden, such as ICU admission or CT scan, were less likely at non–level I trauma centers.

In a study of recommended EBP adherence at 5 level I trauma centers, Shafi et al^43^ identified highly variable adherence to 22 commonly recommended EBPs. Their study found that each 10% increase in institutional adherence to recommended EBPs was associated with reduced mortality; however, the study did not identify factors associated with adherence or evaluate the independent association of each practice with clinical outcomes. In this study, we also identified significant variability in adherence to EBPs. Patients of minority racial/ethnic groups, those who were underinsured, and patients treated at non–level I trauma centers were all less likely to receive EBPs. Patients with less severe injuries (eg, fewer rib fractures) were less likely to receive EBPs. Although the guidelines generally recommend EBPs with specific cutoff levels (eg, Western Trauma Association recommendation that all patients aged S65 years with S3 rib fractures receive ICU care^9^), this finding suggests that clinicians are less likely to follow recommendations for patients with clinical criteria meeting the minimum indication or threshold for an EBP. For example, increased odds of receiving the EBP were seen for patients with indications for ICU admission (7%) and neuraxial blockade (33%) for each additional rib fracture (eTable 2 in the Supplement). This finding is supported by a recent study that noted reduced tertiary trauma center transfer for patients with a near-normal GCS score despite an Advanced Trauma Life Support recommendation that all patients with an abnormal GCS score be transferred to a tertiary trauma center. The authors reported that, despite this recommendation, there was not an overall survival benefit with transfer for patients with a near-normal GCS score.^44^ Future research is needed to clarify EBP unclear areas where adherence to EBP is poor and identify which patient factors derive benefit from EBP and which do not. Randomized clinical trials in trauma can identify novel EBPs that work in general for patients; however, many randomized clinical trials on trauma to date have increased fragility, and poor subgroup analysis is limited in its ability to account for all patient permutations (ie, status changes).^45^ A feasible approach to personalize EBP is to leverage big data analytic techniques to characterize specific patient permutations that benefit. Analysis of big data in this fashion can fuel the development of a framework for a more personalized treatment approach for patients with rib fractures and other traumatic injuries for subsequent validation and optimization within the resources of specific trauma centers. This information can then be leveraged within clinical decision support systems to develop tailored treatment strategies accounting for various patient permutations and adherence that are benchmarked within regional and national quality improvement programs.

Equally important to improving adherence to EBP is the need to evaluate the efficacy of individual EBPs. Mounting evidence suggests that adherence alone to EBPs is a poor marker for high-quality outcomes, raising concerns surrounding the validity of recommended practices.^46^ A study in patients with severe traumatic brain injury identified that only 46% of the patients received recommended EBP and, similar to our study, failed to identify a correlation between institutional adherence to such practices and improved clinical outcomes.^47^

Optimal care for patients with rib fractures is complex and requires engaged, multidisciplinary care. It is possible that centers may develop treatment protocols adhering to EBP measures but poorly integrate and deliver ancillary practices that likely also improve care (eg, multimodal pain therapy, aggressive pulmonary hygiene, and daily ambulation). The delivery of such care pathways for patients with rib fracture has been shown to significantly reduce complication rates and reduce mortality by nearly 3-fold.^19^ Future directions should seek to leverage highly granular electronic health record data repositories to characterize which practices within care pathways are most associated with improved clinical outcomes.

Future work on improving effect estimation for EBP could take the following direction. Instead of additive models implemented in the present study, interaction among putative risk factors could be considered to capture the complex association between the EBP and other patient characteristics to inform heterogeneous treatment outcomes. The selection of interaction terms that enter the final outcome model needs to be implemented in a model selection and performance estimation framework (eg, nested cross-validation) to avoid overfitting.^48^ Similarly, the propensity score model could be improved by incorporating no linear association using more complex models, such as tree-based methods and neural network–based models.^49^

Once effective EBP is better characterized, there is a continued need to improve performance monitoring and EBP adherence. Regional collaboratives have suggested that case mix is not the sole factor involved in clinical outcome variability across institutions, and significant variations in the delivery of EBP and processes of care affects patient outcomes.^50^ Quality improvement initiatives, such as the ACS National Surgical Quality Improvement Program and the ACS Trauma Quality Improvement Program, have fostered significant improvements in EBP by providing institutions with benchmarking reports for patient risk-adjusted process and quality measure adherence, allowing institutions to develop internal quality programs to address specific gaps. These efforts have improved surgical outcomes.^7^ The addition of regional quality collaboratives further improves adherence to EBPs.^51^

To facilitate health care delivery research, there is a need to improve the granularity of data in national registries. The lack of granular details precluded our evaluation of contraindications for certain procedures (eg, neuraxial blockade in the presence of thoracic spine fracture). There are also many reasons why an EBP is not delivered, for example, neuraxial blockade in patients with adequate pain control with oral medication. Thus, it is difficult to ascertain a recommended threshold with which centers should adhere to EBPs. One measure to account for this lack of adherence is to compare hospitals as shown in Figure 2. We identified an association with mortality for patients with hemothorax/pneumothorax who received a tube thoracostomy. However, the *ICD-9* code for hemothorax/pneumothorax does not specify the degree. It is possible that patients with mild hemothorax/pneumothorax were observed and only those with more severe conditions received intervention. Automated data exchange supported by health level 7 standards, a set of standards for transfer of clinical data between software applications,^52,53^ may facilitate the extraction of more granular data from the electronic medical record without adding undue strain on trauma registrars.

### Limitations

This study has several limitations. With respect to the data used in this study, our results may not apply to children (those aged <16 years were excluded), individuals aged 89 years or older (ages recoded for identifiability reasons), or centers with poor quality data (excluded). Another issue is that the measures of injury severity (eg, ISS) may not precisely capture the injury severity information that clinicians use for treatment decisions. This limitation could potentially be overcome by future work leveraging big data to better estimate the severity of a patient’s injury. With respect to the analysis, although we adjusted for confounding risk factors in our models, it is difficult to completely remove the confounding factors as they stem from fundamental issues of selection bias and limitations of the NTDB data elements. We were unable to differentiate physiological vs radiographic-identified flail chest. Thus, we are unable to draw conclusions regarding which population benefits most from rib fixation. Similarly, it is likely that minor procedures, such as chest tube placement, noninvasive ventilation, or CT scans, were not captured by coders. To minimize incomplete coding, this study followed recommended practices for observational data analysis using the NTDB.^11,13^ We also only evaluated the association between these EBPs and mortality, but there are several other outcomes that should be considered, such as complications. With respect to validation, as the NTDB accumulates new data, analysis similar to those applied in this study should be used to monitor the adherence and validate the effectiveness of EBPs.

## Conclusions

Significant variation appears to exist in the delivery of EBPs across US trauma centers for treatment of patients with rib fractures. Only 14% to 42% of patients received EBPs associated with reduced mortality. Multiple factors, including trauma center verification level, appear to be associated with receiving EBPs.

## Supplementary Material

Suppl. materials **eTable 1.** Sample Sizes in Pre- Versus Post-Propensity Matched Cohorts for Each Evidence-Based Practice
**eTable 2.** Patient and Hospital Level Variables Associated With Receiving Evidence Based Practices
**eFigure 1.** Study Diagram Detailing Selection of Patients in 2007–2014 National Trauma Data Bank
**eFigure 2.** EBP1 Propensity Score Distribution Pre (Unmatched) vs Post (Matched)
**eFigure 3.** EBP2 Propensity Score Distribution Pre (Unmatched) vs Post (Matched)
**eFigure 4.** EBP3 Propensity Score Distribution Pre (Unmatched) vs Post (Matched)
**eFigure 5.** EBP4 Propensity Score Distribution Pre (Unmatched) vs Post (Matched)
**eFigure 6.** EBP5 Propensity Score Distribution Pre (Unmatched) vs Post (Matched)
**eFigure 7.** EBP6 Propensity Score Distribution Pre (Unmatched) vs Post (Matched)

## Figures and Tables

**Table 1.** Characteristics of Patients Who Received EBP1 and EBP2^a^

Characteristic	EBP1, No. (%)		*P* value	EBP2, No. (%)		*P* value
	Nonadherence	Adherence		Nonadherence	Adherence	
No.	107 011	4578		65 133	46 456	
Age, median (IQR), y	76 (70–82)	75 (69–82)	.006	76 (69–83)	75 (70–82)	<.001
Male sex	60 095 (56.3)	2592 (56.7)	.59	35 061 (54.0)	27 626 (59.6)	<.001
Race/ethnicity						
White	89 429 (87.2)	4009 (90.6)	<.001	54 855 (87.8)	38 583 (86.7)	<.001
Black	4409 (4.3)	170 (3.8)		2614 (4.2)	1965 (4.4)	
Hispanic	4485 (4.4)	105 (2.4)		2558 (4.1)	2032 (4.6)	
Asian	2186 (2.1)	54 (1.2)		1282 (2.1)	958 (2.2)	
Other	2062 (2.0)	87 (2.0)		1176 (1.9)	973 (2.2)	
Insurance						
Medicaid	2664 (2.7)	88 (2.1)	<.001	1569 (2.6)	1183 (2.8)	<.001
Medicare	61 608 (62.9)	2603 (62.8)		38 756 (65.0)	25 455 (60.0)	
Private	31 354 (32.0)	1402 (33.8)		17 952 (30.1)	14 804 (34.9)	
Self-pay	2244 (2.3)	53 (1.3)		1306 (2.2)	991 (2.3)	
ISS, median (IQR)	14.0 (9.0–21.0)	16.0 (10.0–22.0)	<.001	12.0 (9.0–17.0)	17.0 (13.0–25.0)	<.001
Motor GCS score, median (IQR)	6.0 (6.0–6.0)	6.0 (6.0–6.0)	<.001	6.0 (6.0–6.0)	6.0 (6.0–6.0)	<.001
SBP, median (IQR), mm Hg	142.0 (122.0–161.0)	141.0 (123.0–160.0)	.65	144.0 (126.0–162.0)	139.0 (118.0–159.0)	<.001
Respiratory rate, median (IQR), breaths/min	18.0 (16.0–20.0)	19.0 (16.0–22.0)	<.001	18.0 (16.0–20.0)	18.0 (16.0–22.0)	<.001
Rib fractures, median (IQR), No.	5.0 (4.0–7.0)	7.0 (5.0–9.0)	<.001	5.0 (3.0–6.0)	6.0 (4.0–9.0)	<.001
Hemothorax/pneumothorax	36 057 (33.7)	2261 (49.4)	<.001	18 860 (29.0)	19 458 (41.9)	<.001
ACS-verified level						
I	37 814 (35.3)	1831 (40.0)	<.001	21 030 (32.3)	18 615 (40.1)	<.001
II	20 992 (19.6)	940 (20.5)		13 278 (20.4)	8654 (18.6)	
III	2162 (2.0)	73 (1.6)		1855 (2.8)	380 (0.8)	
IV	46 (<1)	0		34 (0.1)	12 (<1)	
Not distinguished	45 997 (43.0)	1734 (37.9)		28 936 (44.4)	18 795 (40.5)	
Hospital bed size, median (IQR), No.	455 (323–632)	472 (328–632)	.009	450 (319–632)	467 (332–632)	<.001
Admission year						
2007	6236 (5.8)	287 (6.3)	<.001	3921 (6.0)	2602 (5.6)	<.001
2008	9678 (9.0)	367 (8.0)		6063 (9.3)	3982 (8.6)	
2009	11 213 (10.5)	396 (8.7)		6854 (10.5)	4755 (10.2)	
2010	13 041 (12.2)	484 (10.6)		7838 (12.0)	5687 (12.2)	
2011	12 024 (11.2)	520 (11.4)		7245 (11.1)	5299 (11.4)	
2012	16 143 (15.1)	771 (16.8)		9687 (14.9)	7227 (15.6)	
2013	18 015 (16.8)	760 (16.6)		10 892 (16.7)	7883 (17.0)	
2014	20 661 (19.3)	993 (21.7)		12 633 (19.4)	9021 (19.4)	

Abbreviations: ACS, American College of Surgeons; EBP, evidence-based practice; GCS, Glasgow Coma Scale; IQR, interquartile range; ISS, Injury Severity Score; SBP, systolic blood pressure.

a EBP1: neuraxial blockade for patients aged 65 years or older with multiple (≥3) rib fractures; EBP2: intensive care unit admission for patients aged 65 years or older with multiple (≥3) rib fractures.

**Table 2.** Characteristics of Patients Who Received EBP3 and EBP4^a^

Characteristic	EBP3, No. (%)		*P* value	EBP4, No. (%)		*P* value
	Nonadherence	Adherence		Nonadherence	Adherence	
No.	21 017	3302		110 349	1240	
Age, median (IQR), y	54 (44–66)	52 (43–62)	<.001	76 (70–82)	76 (70–82)	.94
Male sex	15 756 (75.1)	2525 (76.6)	.07	61 910 (56.3)	777 (62.7)	<.001
Race/ethnicity						
White	15 880 (79.2)	2559 (80.8)	.03	92 348 (87.3)	1090 (91.3)	<.001
Black	1657 (8.3)	239 (7.5)		4532 (4.3)	47 (3.9)	
Hispanic	1668 (8.3)	232 (7.3)		4568 (4.3)	22 (1.8)	
Asian	290 (1.4)	35 (1.1)		2229 (2.1)	11 (0.9)	
Other	549 (2.7)	104 (3.3)		2125 (2.0)	24 (2.0)	
Insurance						
Medicaid	2217 (11.7)	349 (11.4)	<.001	2739 (2.7)	13 (1.1)	<.001
Medicare	3816 (20.1)	465 (15.2)		63 443 (62.9)	768 (66.3)	
Private	10 193 (53.7)	1850 (60.6)		32 395 (32.1)	361 (31.1)	
Self-pay	2753 (14.5)	391 (12.8)		2280 (2.3)	17 (1.5)	
ISS, median (IQR)	24.0 (17.0–34.0)	25.0 (20.0–34.0)	.005	14.0 (9.0–21.0)	16.0 (10.0–22.0)	<.001
Motor GCS score, median (IQR)	6.0 (4.0–6.0)	6.0 (6.0–6.0)	<.001	6.0 (6.0–6.0)	6.0 (6.0–6.0)	.71
SBP, median (IQR), mm Hg	130.0 (108.0–148.0)	128.0 (110.0–146.0)	.68	142.0 (122.0–160.0)	138.0 (117.0–158.0)	<.001
Respiratory rate, median (IQR), breaths/min	20.0 (16.0–24.0)	20.0 (17.0–25.0)	<.001	18.0 (16.0–20.0)	20.0 (18.0–22.0)	<.001
Rib fractures, median (IQR), No.	NR^b^	NR^b^		5.0 (4.0–7.0)	6.0 (4.0–9.0)	<.001
Hemothorax/pneumothorax	15 747 (74.9)	2763 (83.7)	<.001	37 764 (34.2)	554 (44.7)	<.001
ACS-verified level						
I	8409 (40.0)	1512 (45.8)	<.001	39 220 (35.5)	425 (34.3)	<.001
II	3780 (18.0)	640 (19.4)		21 746 (19.7)	186 (15.0)	
III	223 (1.1)	9 (0.3)		2226 (2.0)	9 (0.7)	
IV	7 (<1)	0		46 (<1)	0	
Not distinguished	8598 (40.9)	1141 (34.6)		47 111 (42.7)	620 (50.0)	
Hospital bed size, median (IQR), No.	448 (321–648)	446 (332–640)	.55	445 (323–632)	515 (370–632)	<.001
Admission year						
2007	1793 (8.5)	120 (3.6)	<.001	6458 (5.9)	65 (5.2)	<.001
2008	2402 (11.4)	151 (4.6)		9968 (9.0)	77 (6.2)	
2009	2487 (11.8)	254 (7.7)		11 506 (10.4)	103 (8.3)	
2010	2744 (13.1)	341 (10.3)		13 378 (12.1)	147 (11.9)	
2011	2278 (10.8)	438 (13.3)		12 422 (11.3)	122 (9.8)	
2012	3006 (14.3)	632 (19.1)		16 703 (15.1)	211 (17.0)	
2013	3022 (14.4)	670 (20.3)		18 551 (16.8)	224 (18.1)	
2014	3285 (15.6)	696 (21.1)		21 363 (19.4)	291 (23.5)	

Abbreviations: ACS, American College of Surgeons; EBP, evidence-based practice; GCS, Glasgow Coma Scale; IQR, interquartile range; ISS, Injury Severity Score; NR, not reported; SBP, systolic blood pressure.

a EBP3: rib fixation for patients with flail chest; EBP4: noninvasive ventilation for patients aged 65 years or older with multiple (≥3) rib fractures.

b For patients with flail chest, the number of rib fractures is not documented in the database.

**Table 3.** Characteristics of Patients Who Received EBP5 and EBP6^a^

Characteristic	EBP5, No. (%)		*P* value	EBP6, No. (%)		*P* value
	Nonadherence	Adherence		Nonadherence	Adherence	
No.	149 174	109 160		49 012	32 405	
Age, mean (SD), y	48 (32–61)	48 (31–61)	<.001	76 (70–83)	76 (69–82)	<.001
Male sex	105 136 (70.5)	81 590 (74.8)	<.001	26 629 (54.5)	18 124 (56.0)	<.001
Race/ethnicity						
White	109 065 (75.9)	77 298 (73.5)	<.001	39 565 (84.8)	27 481 (87.6)	<.001
Black	14 150 (9.8)	13 909 (13.2)		2287 (4.9)	1438 (4.6)	
Hispanic	13 704 (9.5)	9671 (9.2)		2375 (5.1)	1295 (4.1)	
Asian	2320 (1.6)	1279 (1.2)		1392 (3.0)	560 (1.8)	
Other	4441 (3.1)	2987 (2.8)		1057 (2.3)	587 (1.9)	
Insurance						
Medicaid	15 929 (12.1)	14 229 (14.3)	<.001	1095 (2.5)	832 (2.7)	<.001
Medicare	21 161 (16.0)	15 421 (15.5)		28 198 (63.7)	18 494 (60.4)	
Private	72 601 (55.0)	51 884 (52.0)		13 808 (31.2)	10 681 (34.9)	
Self-pay	22 312 (16.9)	18 278 (18.3)		1193 (2.7)	591 (1.9)	
ISS, median (IQR)	17.0 (12.0–24.0)	21.0 (14.0–29.0)	<.001	13.0 (9.0–21.0)	14.0 (9.0–20.0)	<.001
Motor GCS score, median (IQR)	6.0 (6.0–6.0)	6.0 (5.0–6.0)	<.001	6.0 (6.0–6.0)	6.0 (6.0–6.0)	<.001
SBP, median (IQR), mm Hg	134.0 (118.0–150.0)	129.0 (109.0–146.0)	<.001	143.0 (123.0–162.0)	144.0 (124.0–163.0)	<.001
Respiratory rate, median (IQR), breaths/min	18.0 (16.0–22.0)	20.0 (16.0–24.0)	<.001	18.0 (16.0–20.0)	18.0 (16.0–21.0)	<.001
Rib fractures, median (IQR), No.	4.0 (2.0–7.0)	5.0 (2.0–8.0)	<.001	5.0 (4.0–7.0)	5.0 (4.0–8.0)	<.001
Hemothorax/pneumothorax	149 174 (100)	109 160 (100)	NA	14 853 (30.3)	11 422 (35.2)	<.001
ACS-verified level						
I	56 528 (37.9)	42 813 (39.2)	<.001	14 688 (30.0)	10 847 (33.5)	<.001
II	28 454 (19.1)	18 852 (17.3)		10 774 (22.0)	6157 (19.0)	
III	1935 (1.3)	1142 (1.0)		1399 (2.9)	726 (2.2)	
IV	100 (0.1)	36 (<1)		41 (0.1)	5 (<1)	
Not distinguished	62 157 (41.7)	46 317 (42.4)		22 110 (45.1)	14 670 (45.3)	
Hospital bed size, median (IQR), No.	455 (324–640)	467 (333–640)	<.001	446 (317–620)	455 (321–600)	<.001
Admission year						
2007	11 392 (7.6)	8349 (7.6)	<.001	3677 (7.5)	1160 (3.6)	<.001
2008	15 861 (10.6)	11 515 (10.5)		5561 (11.3)	1987 (6.1)	
2009	18 237 (12.2)	11 601 (10.6)		6103 (12.5)	2350 (7.3)	
2010	19 148 (12.8)	13 541 (12.4)		6691 (13.7)	3216 (9.9)	
2011	16 254 (10.9)	13 310 (12.2)		5488 (11.2)	3646 (11.3)	
2012	22 205 (14.9)	16 168 (14.8)		7033 (14.3)	5309 (16.4)	
2013	22 153 (14.9)	16 691 (15.3)		7139 (14.6)	6464 (19.9)	
2014	23 924 (16.0)	17 985 (16.5)		7320 (14.9)	8273 (25.5)	

Abbreviations: ACS, American College of Surgeons; EBP, evidence-based practice; GCS, Glasgow Coma Scale; IQR, interquartile range; ISS, Injury Severity Score; NA, not applicable; SBP, systolic blood pressure.

a EBP5: chest tube placement for patients with hemothorax/pneumothorax; EBP6: chest tomographic scan for patients aged 65 years or older with multiple (≥3) rib fractures.

## Key Points

### Question

What are the adherence to evidence-based practice for patients with rib fractures across US trauma centers and its association with mortality?

### Findings

In this cohort study of 625 617 patients with traumatic rib fractures, significant variation existed in the delivery of evidence-based practices for rib fractures across US trauma centers. Three evidence-based practices were associated with reduced mortality, but adherence was poor; multiple factors, including trauma center verification level, were associated with receiving evidence-based practices for rib fractures.

### Meaning

Variability appears to exist in the delivery and outcomes of evidence-based practice, and future research performance monitoring directions or efforts should use granular data to characterize practices that improve clinical outcomes.
