# Supplementary material for: Association Between Adherence to Evidence-Based Practices for Treatment of Patients With Traumatic Rib Fractures and Mortality Rates Among US Trauma Centers
Source: JAMA Netw Open. Author manuscript; Available in PMC 2021 Mar 2. (PMC7707110; doi:10.1001/jamanetworkopen.2020.1316)
Supplement: Suppl. materials — eTable 1. Sample Sizes in Pre- Versus Post-Propensity Matched Cohorts for Each Evidence-Based Practice eTable 2. Patient and Hospital Level Variables Associated With Receiving Evidence Based Practices eFigure 1. Study Diagram Detailing Selection of Patients in 2007–2014 National Trauma Data Bank eFigure 2. EBP1 Propensity Score Distribution Pre (Unmatched) vs Post (Matched) eFigure 3. EBP2 Propensity Score Distribution Pre (Unmatched) vs Post (Matched) eFigure 4. EBP3 Propensity Score Distribution Pre (Unmatched) vs Post (Matched) eFigure 5. EBP4 Propensity Score Distribution Pre (Unmatched) vs Post (Matched) eFigure 6. EBP5 Propensity Score Distribution Pre (Unmatched) vs Post (Matched) eFigure 7. EBP6 Propensity Score Distribution Pre (Unmatched) vs Post (Matched) [file NIHMS1639520-supplement-Suppl__materials.pdf]

Supplementary Online Content

Tignanelli CJ, Rix A, Napolitano LM, Hemmila MR, Ma S, Kummerfeld E. Association between adherence to evidence-based practices for treatment of patients with traumatic rib fractures and mortality rates among US trauma centers. *JAMA Netw Open*. 2020;3(3):e201316. doi:10.1001/jamanetworkopen.2020.1316

eTable 1. Sample Sizes in Pre- Versus Post-Propensity Matched Cohorts for Each Evidence-Based Practice

eTable 2. Patient and Hospital Level Variables Associated With Receiving Evidence Based Practices

eFigure 1. Study Diagram Detailing Selection of Patients in 2007-2014 National Trauma Data Bank

eFigure 2. EBP1 Propensity Score Distribution Pre (Unmatched) vs Post (Matched)

eFigure 3. EBP2 Propensity Score Distribution Pre (Unmatched) vs Post (Matched)

eFigure 4. EBP3 Propensity Score Distribution Pre (Unmatched) vs Post (Matched)

eFigure 5. EBP4 Propensity Score Distribution Pre (Unmatched) vs Post (Matched)

eFigure 6. EBP5 Propensity Score Distribution Pre (Unmatched) vs Post (Matched)

eFigure 7. EBP6 Propensity Score Distribution Pre (Unmatched) vs Post (Matched)

This supplementary material has been provided by the authors to give readers additional information about their work.

© 2020 Tignanelli CJ et al. *JAMA Network Open.*

eTable 1: Sample sizes in pre- versus post-propensity matched cohorts for each evidence-based practice

	**PRE-<br>EBP adherence**	**PRE-<br>EBP non-adherence**	**Matched POST-<br>EBP adherence**	**Matched POST-<br>EBP non-adherence**
EBP1	77398	3661	3661	3661
EBP2	40859	40213	23097	40213
EBP3	20225	3243	3243	3243
EBP4	34178	986	986	986
EBP5	125481	96279	85156	85156
EBP6	32924	24105	24092	24092

Abbreviation: EBP, evidence-based practice

© 2020 Tignanelli CJ et al. *JAMA Network Open.*

eTable 2: Patient and hospital level variables associated with receiving evidence based practices

	Epidural		ICU Admission		Rib Fixation		NIV		Chest Tube		CT Chest	
	OR	p-value	OR	p-value	OR	p-value	OR	p-value	OR	p-value	OR	p-value
**Demographics**												
Age	0.998	0.35	1.01	<0.001	0.99	<0.001	1.01	0.23	1.005	<0.001	0.997	0.009
Male	0.92	0.027	1.13	<0.001	1.04	0.40	1.21	0.007	1.30	<0.001	1.01	0.66
Race												
White	(ref)											
Black	0.91	0.28	1.03	0.37	0.84	0.020	0.89	0.47	1.37	<0.001	0.89	0.007
Hispanic	0.49	<0.001	1.05	0.22	0.80	0.003	0.58	0.025	0.92	<0.001	0.74	<0.001
Asian	0.58	<0.001	1.03	0.62	0.75	0.11	0.74	0.35	0.77	<0.001	0.51	<0.001
Other	0.96	0.73	0.99	085	1.14	0.22	0.97	0.90	0.89	<0.001	0.79	<0.001
Insurance												
Medicaid	(ref)											
Medicare	1.24	0.058	1.19	<0.001	0.92	0.29	1.54	0.10	0.92	<0.001	0.91	0.074
Private	1.18	0.15	1.23	<0.001	1.13	0.043	1.46	0.16	0.87	<0.001	0.97	0.57
Self-pay	0.70	0.052	0.88	0.046	0.91	0.23	1.14	0.72	0.93	<0.001	0.71	<0.001
ICU Admission	1.79	<0.001	N/A		1.43	<0.001	1.81	<0.001	1.22	<0.001	1.26	<0.001
**Injury Severity**												
ISS	0.98	<0.001	1.03	<0.001	1.00	0.83	0.99	0.003	1.01	<0.001	0.98	<0.001
SBP	1.00	<0.001	1.00	0.031	1.00	0.82	1.00	0.003	0.99	<0.001	1.00	<0.001
Respiratory Rate	1.00	0.60	1.02	<0.001	1.01	<0.001	1.01	0.017	1.02	<0.001	1.00	0.033
Pulse	1.00	0.031	1.01	<0.001	1.00	<0.001	1.01	0.002	1.01	<0.001	1.00	0.000
GCS Motor	1.32	<0.001	0.92	<0.001	1.08	<0.001	1.07	0.017	0.89	<0.001	1.04	0.000
# of Rib Fracture	1.33	<0.001	1.07	<0.001	N/A		1.07	<0.001	1.01	<0.001	1.07	<0.001
Flail Chest	0.88	0.022	0.95	0.16	N/A		1.07	0.59	1.80	<0.001	0.88	0.001
Hemopneumothorax	1.52	<0.001	1.21	<0.001	1.51	<0.001	1.22	0.004	NA		1.14	<0.001
**Hospital Features**												
Verification Level												
ACS Level 1	(ref)											
ACS Level 2	1.09	0.089	0.87	0.000	0.92	0.13	1.34	0.008	0.97	0.032	0.74	<0.001
ACS Level 3	0.99	1.0	0.38	<0.001	0.24	<0.001	1.76	0.13	1.08	0.09	0.83	0.006
ACS Level 4			0.77	0.54					0.57	0.025	0.16	0.003
Undesignated	0.88	0.002	0.83	<0.001	0.72	<0.001	1.41	<0.001	1.03	0.004	0.86	<0.001
Hospital Bed Size	1.0003	0.001	0.9998	<0.001	0.9998	0.017	1.0002	0.29	1.0001	<0.001	0.9998	<0.001

Abbreviations: ICU, intensive care unit, ISS, injury severity score, SBP, systolic blood pressure, GCS, Glasgow Coma Scale

© 2020 Tignanelli CJ et al. *JAMA Network Open.*

eFigure 1: Study diagram detailing selection of patients in 2007-2014 National Trauma Data Bank

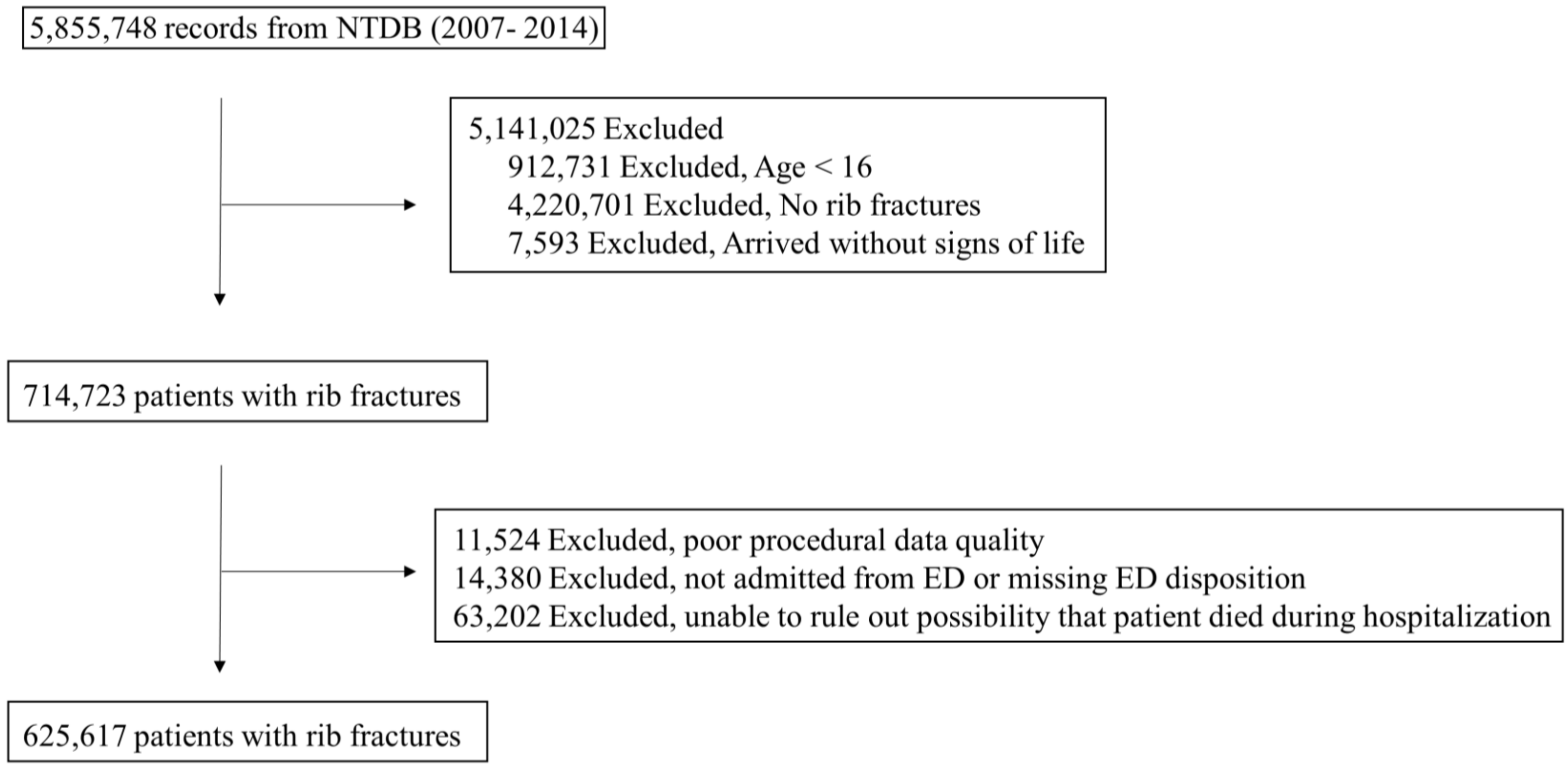

© 2020 Tignanelli CJ et al. *JAMA Network Open.*

eFigure 2: EBP1 propensity score distribution Pre (unmatched) vs post (matched).

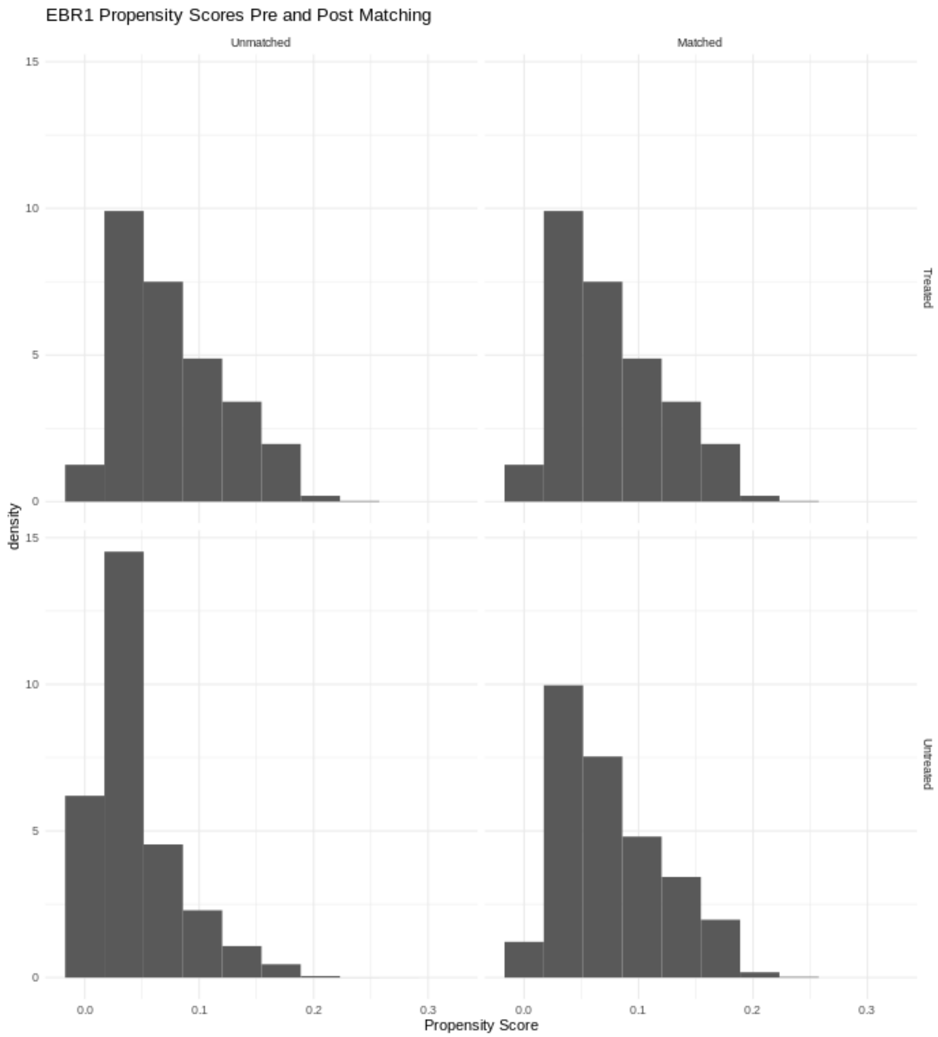

© 2020 Tignanelli CJ et al. *JAMA Network Open.*

eFigure 3: EBP2 propensity score distribution Pre (unmatched) vs post (matched).

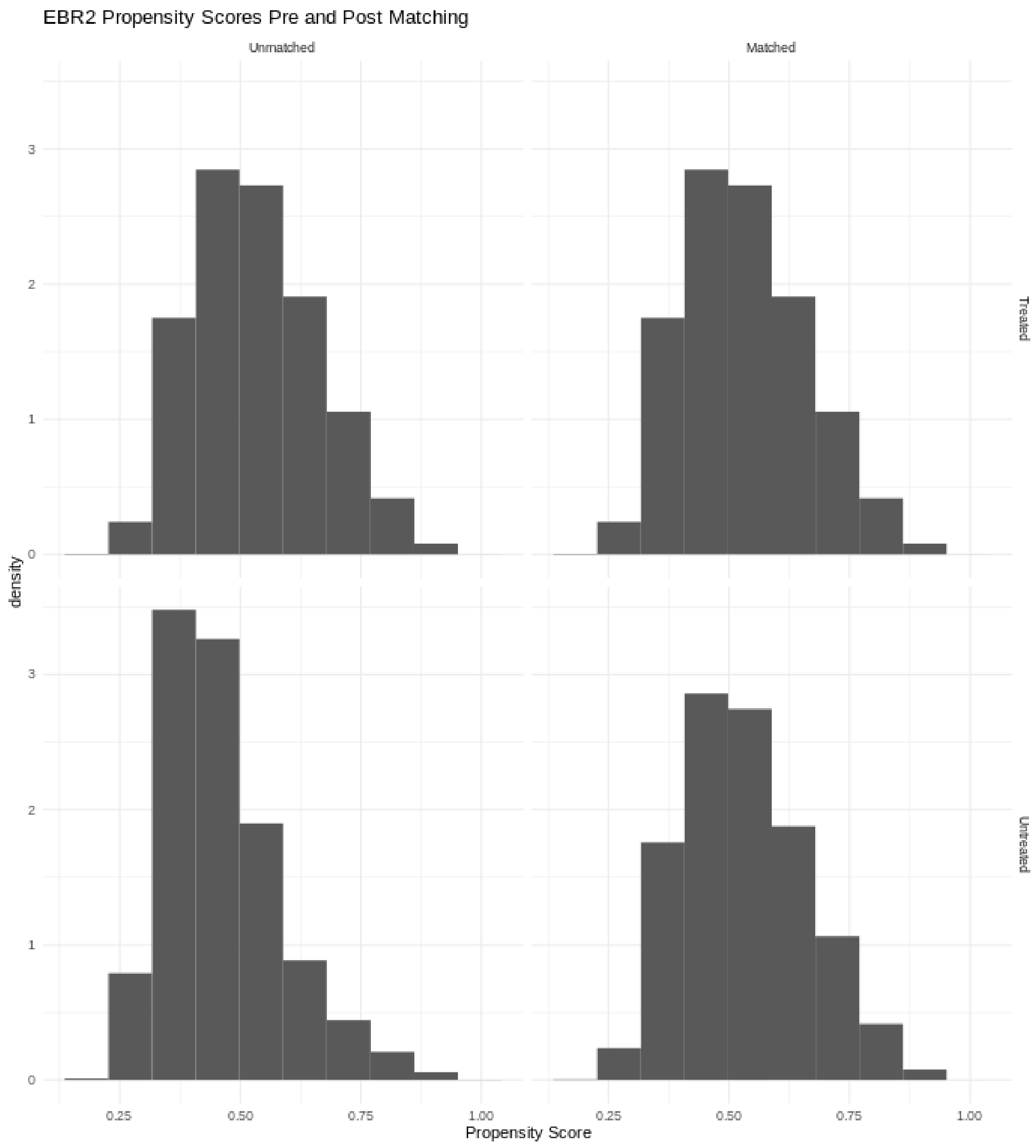

© 2020 Tignanelli CJ et al. *JAMA Network Open.*

e Figure 4: EBP3 propensity score distribution Pre (unmatched) vs post (matched).

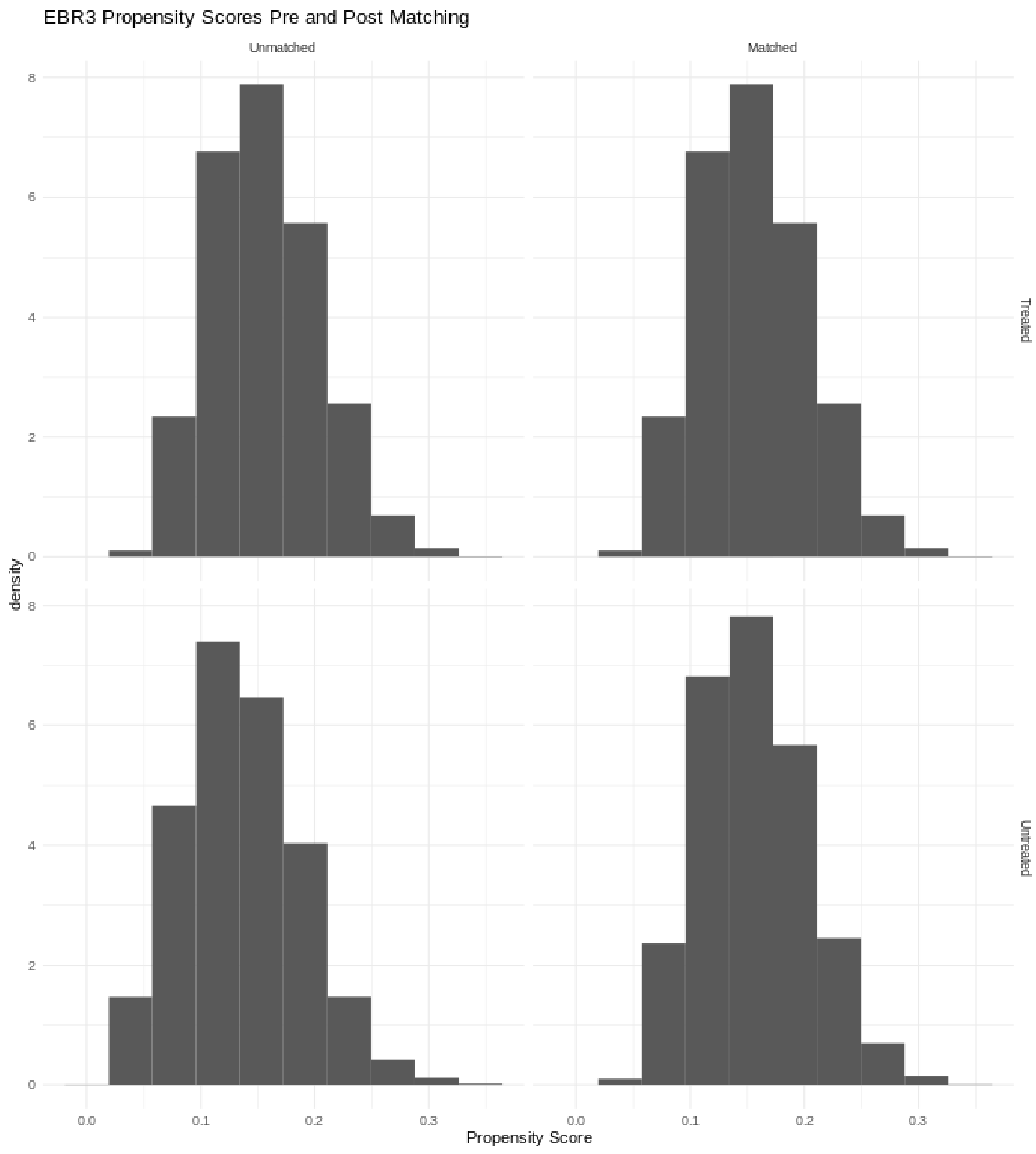

© 2020 Tignanelli CJ et al. *JAMA Network Open.*

eFigure 5: EBP4 propensity score distribution Pre (unmatched) vs post (matched).

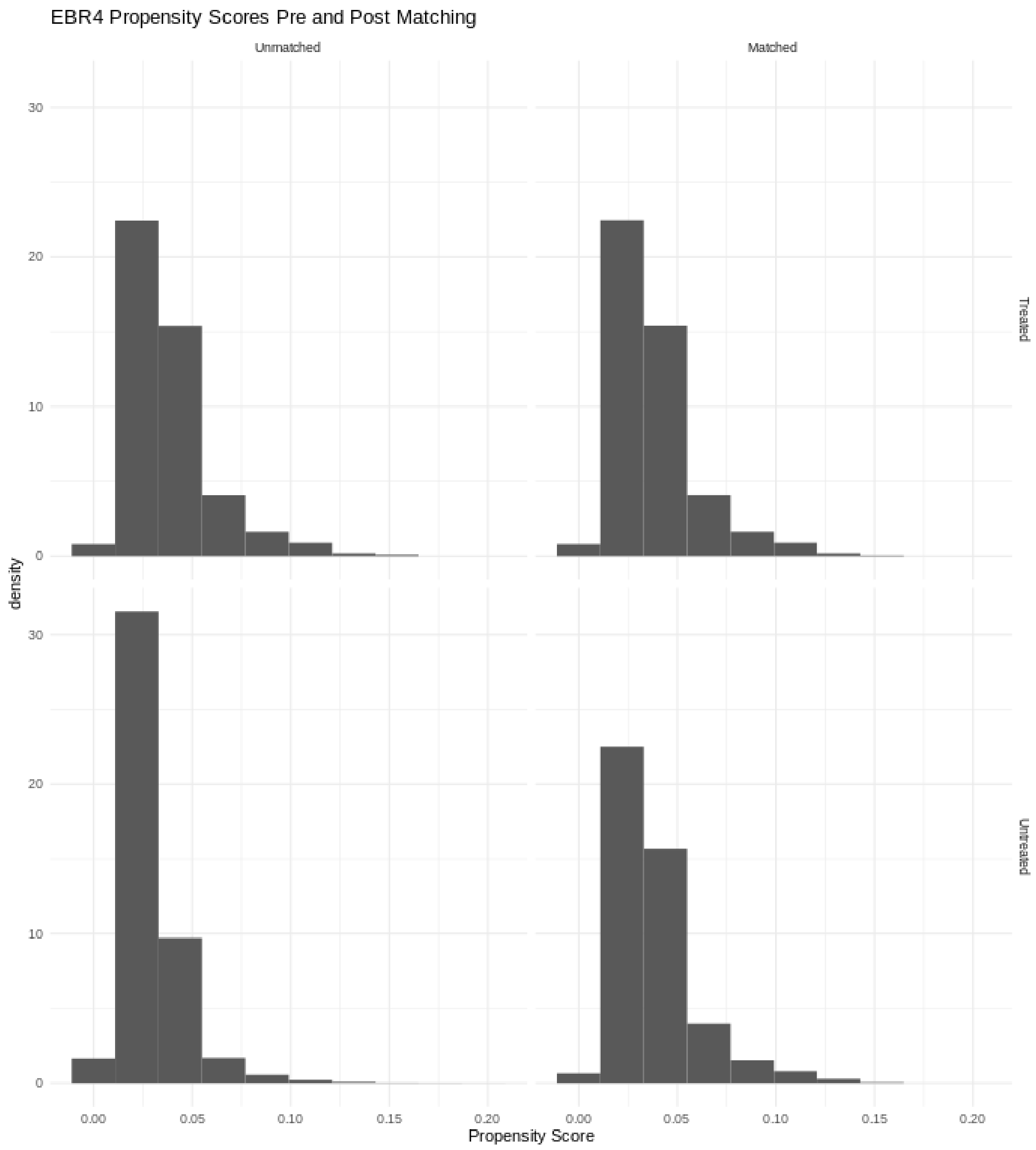

© 2020 Tignanelli CJ et al. *JAMA Network Open.*

eFigure 6: EBP5 propensity score distribution Pre (unmatched) vs post (matched).

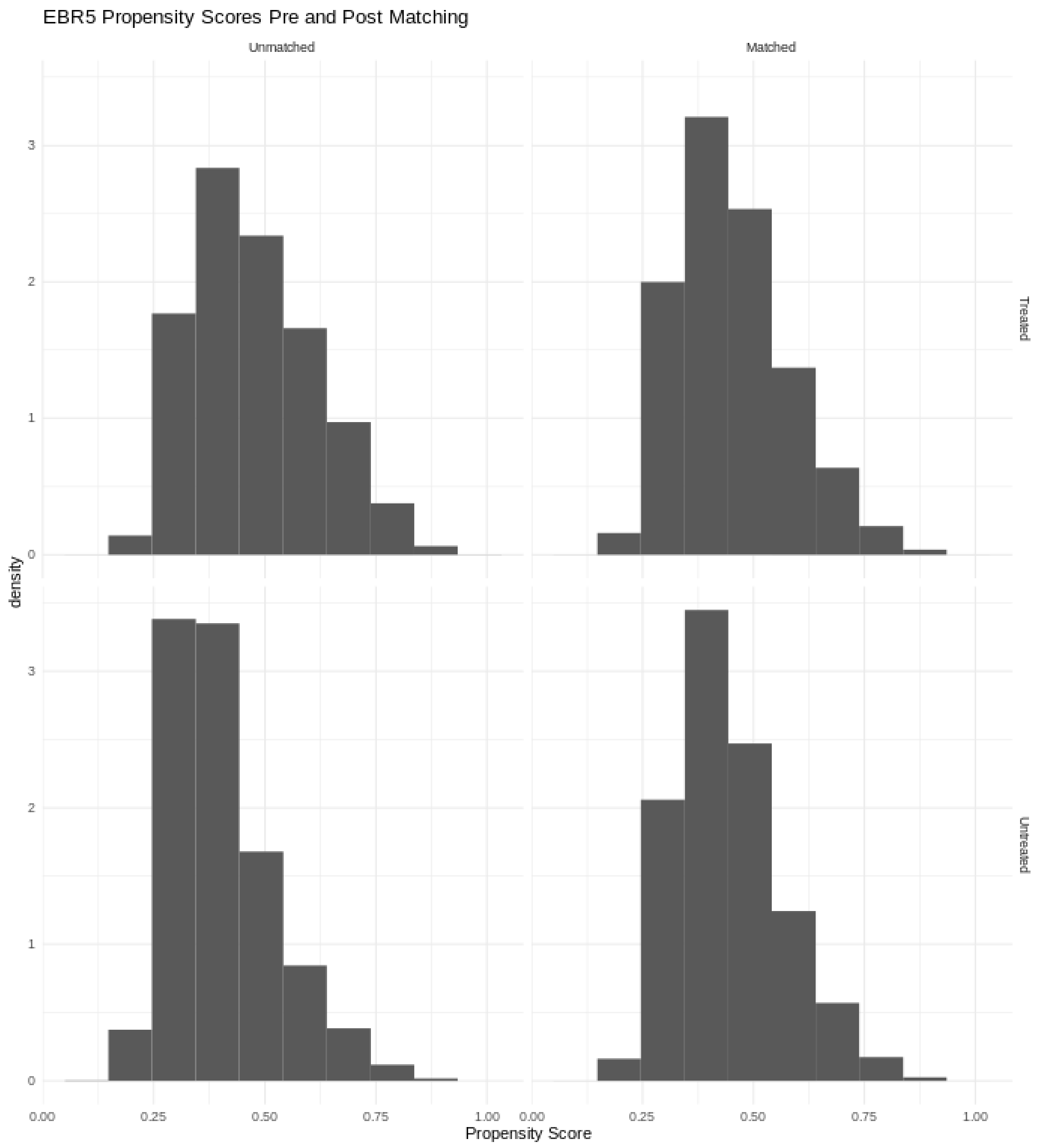

© 2020 Tignanelli CJ et al. *JAMA Network Open.*

e Figure 7: EBP6 propensity score distribution Pre (unmatched) vs post (matched).

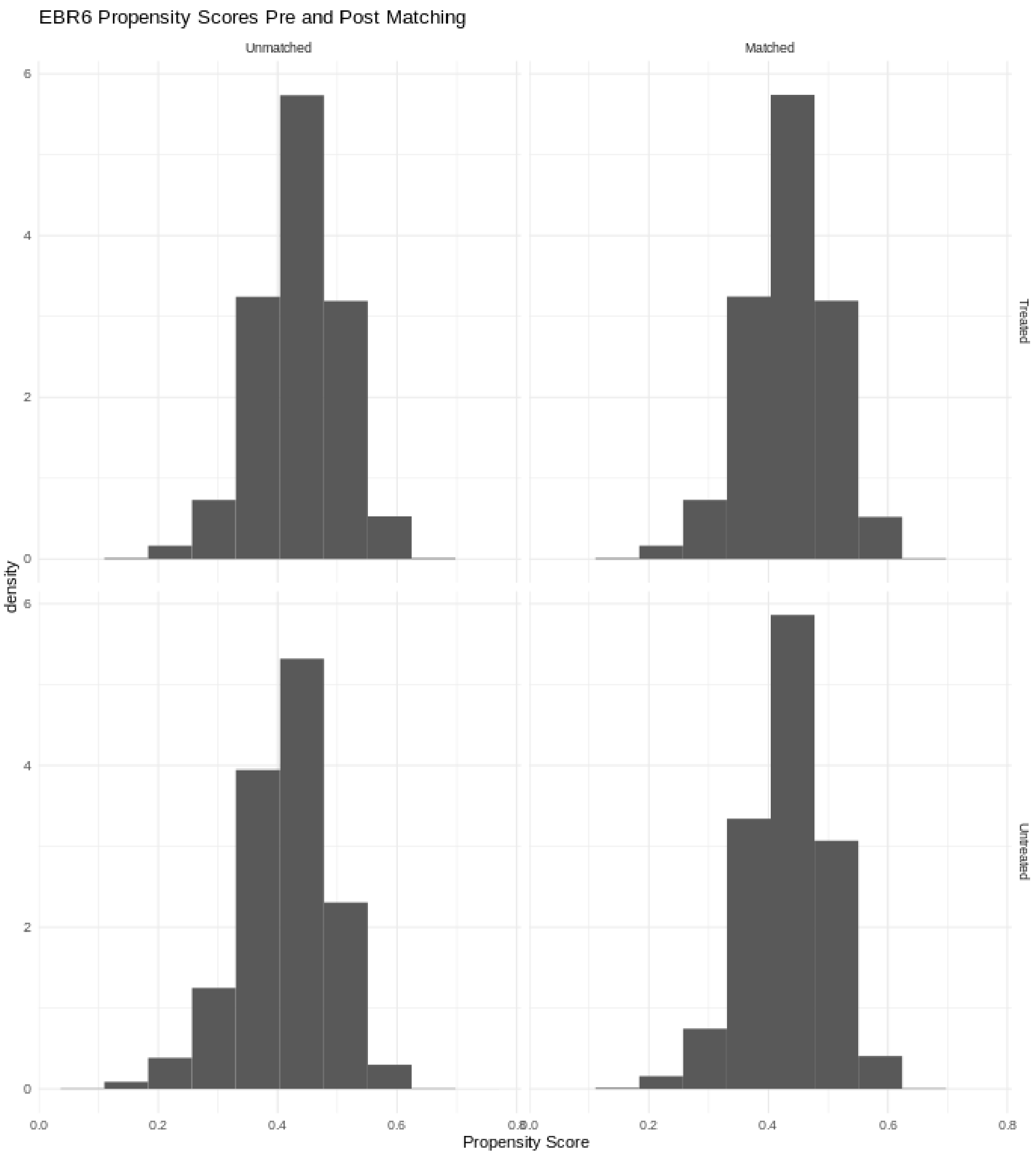

© 2020 Tignanelli CJ et al. *JAMA Network Open.*
